# Rapid growth accelerates telomere attrition in a transgenic fish

**DOI:** 10.1186/s12862-015-0436-8

**Published:** 2015-08-14

**Authors:** Angela Pauliny, Robert H. Devlin, Jörgen I. Johnsson, Donald Blomqvist

**Affiliations:** Department of Biological and Environmental Sciences, University of Gothenburg, Box 463, 405 30 Gothenburg, Sweden; Marine Ecosystems and Aquaculture Division, Fisheries and Oceans Canada, 4160 Marine Drive, West Vancouver, BC V7V 1N6 Canada

## Abstract

**Background:**

Individuals rarely grow as fast as their physiologies permit despite the fitness advantages of being large. One reason may be that rapid growth is costly, resulting for example in somatic damage. The chromosomal ends, the telomeres, are particularly vulnerable to such damage, and telomere attrition thus influences the rate of ageing. Here, we used a transgenic salmon model with an artificially increased growth rate to test the hypothesis that rapid growth is traded off against the ability to maintain somatic health, assessed as telomere attrition.

**Results:**

We found substantial telomere attrition in transgenic fish, while maternal half-sibs growing at a lower, wild-type rate seemed better able to maintain the length of their telomeres during the same time period.

**Conclusions:**

Our results are consistent with a trade-off between rapid growth and somatic (telomere) maintenance in growth-manipulated fish. Since telomere erosion reflects cellular ageing, our findings also support theories of ageing postulating that unrepaired somatic damage is associated with senescence.

**Electronic supplementary material:**

The online version of this article (doi:10.1186/s12862-015-0436-8) contains supplementary material, which is available to authorized users.

## Background

Growing to a large body size often provides selective advantages, both in terms of an individual’s own survival as well as its fecundity. Therefore, one would expect individuals to grow as fast as they can. Such maximum growth rates are rarely observed, however, suggesting that rapid growth is costly [1, 2]. Generally, trade-offs in nature arise from energy and time constraints, and an individual is expected to allocate a finite amount of resources to various processes in such a way as to maximise its fitness. Indeed, several studies have documented a link between growth rate and lifespan (e.g. [3]; reviewed in [4]). In addition, the phenotypic effects of growth hormone (GH) transgenesis in fish and mice include shorter lifespans compared to wild-type controls, further supporting a trade-off between investments in growth and longevity assurance measures (e.g. [5–7]). However, the mechanistic links behind this trade-off are far from understood.

Phenotypic growth, the increase in body size due to cell proliferation, requires activation of DNA and protein synthesis. The by-products of aerobic metabolism are free radicals, such as reactive oxygen species (hereafter ROS), which have important functions as cell signalling molecules [8, 9]. However, when produced in excess, e.g. during rapid growth, they result in oxidative stress and damage macromolecules including DNA, proteins and lipids [8–10]. The free radical theory of ageing [11] postulates that ageing processes are governed by the accumulation of unrepaired somatic damage incurred by free radicals. An elevated growth rate may therefore result in accelerated ageing and a shortened lifespan (e.g. [12]).

To enable a longer intrinsic lifespan, an organism relies on an extensive suite of maintenance mechanisms including DNA and protein repair, defences against ROS, apoptosis, immune response, and wound healing [13]. In particular, telomeres and their maintenance have emerged as an important factor influencing the rate of cellular and organismal senescence (e.g. [14–16]). These dynamic nucleoprotein structures at the end of eukaryotic chromosomes have a multitude of vital functions. For example, a telomere and its associated shelterin complex promote genome stability [17], and play an important role in modifying the expression of subtelomeric genes [18]. Because conventional DNA polymerases are unable to complete the replication of the lagging strand, telomeres shorten at a slow pace with each replicative cell cycle (the so called end-replication problem; [19]). Numerous empirical studies have thus documented a negative relationship between age and telomere length of cells or individuals (e.g. [14, 20]). In addition, the sequence of telomeric DNA is particularly prone to attack from ROS [21, 22]. Consistently, oxidative stress was shown to cause an increased number of single-strand breaks leading to the loss of distal telomere fragments and accelerated telomere attrition [23]. This mechanism of telomere shortening may therefore be more important for the rate of ageing than incomplete replication [23]. Since only sufficiently long telomeres are able to exert their functions, cells may express the enzyme telomerase to counteract various telomere-shortening factors [24, 25]. This ribonucleoprotein enzyme uses an integrated RNA template to specifically elongate telomeric DNA at chromosome termini [15].

In birds and mammals, telomere attrition is slower in long-lived species than in short-lived ones (e.g. [20, 26, 27]). Moreover, individuals with relatively long telomeres for their age have higher fitness, including greater longevity (e.g. [20, 28, 29]). Fishes have evolved a large variety of life-history strategies, and most fish species continue to grow throughout life [30]. In contrast to humans, somatic cells in fish express significant levels of telomerase (reviewed in [31]), suggesting improved telomere length maintenance as a possible mechanism behind indeterminate growth. Indeed, also the remarkable ability of fish to regenerate rayed fins and other organs may be achieved through upregulation of telomerase expression [32, 33], or through activation of local stem cells (reviewed in [34]) that have retained an individual’s initial telomere length [25].

With a fast increasing human population while wild fish stocks are dwindling, transgenic technologies have been explored in the hope to improve aquaculture food production efficiency and yield of commercially important fish species. For example, substantially faster growth rates in GH-transgenic salmons are achieved by stable incorporation of a constitutively expressed GH gene construct into the fish’s genome [35–37]. The effects of enhanced growth in the semelparous coho salmon (*Oncorhynchus kisutch*) are apparent throughout life and include significantly earlier hatching of larger and heavier fry, earlier onset of smoltification and sexual maturation, and a shortened lifespan [6]. This “live life in the fast lane” phenotype therefore follows predictions from the free radical theory of ageing, making growth-enhanced transgenic fish a suitable model to study mechanisms behind the ageing process as well as costs of rapid growth.

Using a paired design (i.e. analysing repeat samples of the same individual), we tested the hypothesis that growth rate affects telomere maintenance, potentially influencing the rate of cellular ageing. We thus examined whether GH-transgenic fish with a markedly increased growth rate compared to wild-type maternal half-sibs were (1) unable to maintain the length of their telomeres, and (2) consequently had shorter telomeres in non-regenerated fins compared to equivalent regenerated fins (since the telomeres of the latter should have been restored, as argued above).

## Methods

### GH-transgenic salmon strain and fish husbandry

We studied growth–manipulated coho salmon (*Oncorhynchus kisutch*), a semelparous species in which individuals die at maturation [38]. The transgene utilized in the present experiments, OnMTGH1, constitutively drives elevated levels of GH from the metallothionein-B promoter and causes strongly enhanced growth rates [6, 36]. The strain of transgenic salmon used (M77) was originally generated, and has been subsequently maintained, in a wild genetic background using fish obtained from nature (Chehalis River, British Columbia). We produced GH-transgenic fish by crossing ten homozygous transgenic males (randomly selected from a stock population) to half the eggs from ten wild-type Chehalis River females. Wild-type salmons were generated by crossing ten wild-type Chehalis River males to the remaining eggs from each female. Thus, transgenic offspring contain a single copy of the GH-transgene but otherwise possess the same wild-type (Chehalis River) genetic background as their non-transgenic half-sibs. The transgenic genotype of experimental animals was verified using a transgene-specific PCR assay as described previously [6].

Wild-type and GH-transgenic salmon families were reared in separate tanks (mixed sex) of approximately 4000 individuals each to avoid interaction effects arising from the very different feeding motivations and growth rates between the genotypes. At all times, the density of fish was kept below 5 kg m^−3^. Tanks were supplied with fresh well water (10 °C) and aeration, and were exposed to natural photoperiods using simulated daylight illumination. Fish were fed to satiation twice daily with commercial salmon feeds (Skretting Canada). The size of pellets offered was appropriate for the body size (stage) of the fish, and were separately adjusted throughout the study period as the animals grew.

### Sampling procedures

**Fig. 1:**
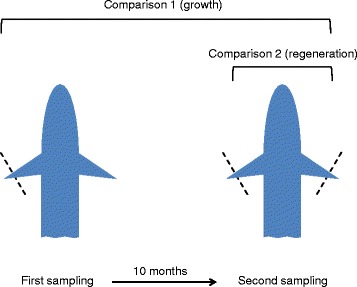
Schematic of sampling procedure. Each individual was sampled twice. Telomere lengths in clipped pelvic fins were compared to assess effects of growth (comparison 1) or regeneration (comparison 2) on within-individual telomere attrition

On 23 July 2009, at the age of 28 weeks postfertilization, we randomly selected and pit-tagged 30 GH-transgenic and 30 wild-type half-sibs, and collected the first small sample of the left pelvic fin from each individual. Due to the haphazard selection of animals, we do not know the family of origin of selected fish (but see Data processing and statistical analyses, below). After about 10 months of growth (307 days), we re-sampled all individuals that were still alive on 26 May 2010 by clipping both their pelvic fins (Fig. 1). All fin samples were snap-frozen in liquid nitrogen and stored in 96 % ethanol at –80 °C for 1–2 years until further analyses.

Our data available for analyses consisted of 23 transgenic and 15 wild-type fish. Some of the originally marked fish were lost due to PIT tag malfunction (*n* = 4) or bacterial infection resulting in termination of the individual (*n* = 3), respectively. In the remaining cases (*n* = 15), we do not know the reason why fish died. There was, however, no significant difference in the frequency of losses between transgenic and wild-type fish (χ^2^ = 1.51, df = 1, *p* = 0.22; excluding cases of technical errors).

Fish were measured and weighed at both sampling occasions. Weight, length, and specific growth rates (SGR) for each genotype are presented in Additional file 1.

All procedures were approved by the Fisheries and Oceans Canada Pacific Region Animal Care Committee (permit AUP 09-009) and met guidelines outlined by the Canadian Council for Animal Care.

### Telomere length assay

Relative telomere length was measured using quantitative real-time PCR (qPCR; [39]), and applicable MIQE guidelines [40] were followed to ensure a high level of quality and transparency in our qPCR analyses. We used our previously described qPCR assays for brown trout *Salmo trutta* [41], which also yielded efficient and specific amplification in the related coho salmon (see below for details).

Genomic DNA was freshly extracted from fin clips using the DNeasy Blood & Tissue Kit (Qiagen) and the manufacturer’s protocol. DNA quantity and quality were assessed with a NanoDrop spectrophotometer. Only samples with a minimum A_260/280_ of 1.8 were included in the study (mean ± SE = 2.1 ± 0.005, *n* = 114). After preparing a working stock of 10 ng μl^−1^ in autoclaved and aliquoted purified water (Milli-Q; hereafter water), their concentrations were re-analysed and diluted with water to a final sample concentration of 0.5 ng μl^−1^.

Before quantifying telomeric content, each sample was tested in duplicate for qPCR inhibitors (e.g. remains of ethanol) using the SPUD assay [42]. The threshold cycle numbers (Cq) of the SPUD spike amplified in water or with one of our samples present was comparable, thus showing no marked effect of inhibitors on the efficiency of qPCR reactions [42].

Quantitative real-time PCR records the accumulating fluorescent signal as amplification of the target DNA proceeds [43]. For each sample, the fractional cycle number at which the signal reaches a set threshold above baseline fluorescence (Cq) is determined. Thus, the Cq value of a sample is inversely proportional to the starting amount of template DNA, e.g. telomere repeats. Telomeric content per cell (genome), a proxy for telomere length (e.g. [39]), was determined as the number of telomere repeats (T) per number of reference gene copies (S). Building on Cawthon [39], we derived a relative measure of individual telomere length by comparing (i.e. standardizing) the T/S ratio of each focal sample to that of a calibrator sample (included on all plates). We used beta-actin (hereafter actin) as a reference gene. Forward and reverse actin primers were designed in Beacon Designer (PREMIER Biosoft) based on the published beta-actin mRNA sequence of a related salmon species (*Oncorhynchus mykiss*, [GenBank:AF157514]). For amplification of telomeric repeats, universal primers were used (for all primer sequences see Additional file 2). Both PCR reactions were optimised using the machine’s gradient function, and amplicon size as well as specificity was confirmed by agarose gel electrophoresis (data not shown). Each qPCR reaction contained 4 ng DNA in a total volume of 20 *μ*l 1x KAPA SYBR Fast Mastermix (2.5 mM final MgCl_2_, KAPA Biosystems). Final concentrations of forward and reverse primers for the telomere amplification were 100 nM and 200 nM, respectively, whereas 350 nM was used for each of the actin primers. Reactions were set up manually and amplified on a BioRad iCycler qPCR machine using FrameStar PCR plates (4titude). Reaction conditions included an initial denaturation at 95 °C for 4 min, followed by 25 (telomere) or 40 (actin) cycles of 95 °C for 15 s and 56 °C for 1 min. After each run was completed, a melt curve (55 °C - 95 °C, 0.5 °C increase cycle^−1^) was generated to assess PCR specificity. Corresponding telomere and actin amplifications were carried out on different plates (but same well position) and right after each other on the same day, using aliquots from the same preparation of sample dilutions.

All experimental samples were analysed in triplicate, and average values were used in the subsequent analyses. In 4 out of 228 cases (1.8 %), one of the triplicates deviated markedly from the other two measurements (more than 1 amplification cycle) and was therefore conservatively excluded in subsequent analyses. Individuals were randomly assigned to one of five plates, but repeat samples from the same individual were analysed on the same plate for optimal comparison. Thus, we refrained from analysing samples from individual fish across plates. The intra-plate coefficient of variation (samples run in triplicate) ranged between 0.17 % - 3.19 % (telomere), and 0.042 % - 2.35 % (actin). To estimate the amplification efficiency of each plate, a standard curve consisting of 5 serial 1:10 dilutions of one sample was analysed in triplicate (10,000-fold range, 50 ng - 0.005 ng DNA per well, with the middle quantity roughly matching that of samples being analysed). Standard curves were generated by the iQ5 software v. 2.0 (BioRad), and PCR efficiencies (E) calculated as E = 10 ^[–1/slope]^. PCR efficiencies were generally high in the investigated range (50 ng - 0.005 ng), as was the linearity of the model (all R^2^ > 0.985). Standard curve characteristics (slope, y-intercept and R^2^) as well as E of all plates are presented in Additional file 3. On each plate, a negative control (no template control, NTC) was included in triplicate. The Cq of NTCs for the telomere and actin amplifications was at least 6 and 5 cycles higher, respectively, than with template present (mean difference, telomere: 10.04 cycles; actin: 9.6 cycles). Thus, fluorescence signals derived from samples were approximately 1024 times stronger than background noise (E^cycles^ = 2^10^ = 1024), assuming a PCR efficiency of 100 % (E = 2, reflecting a perfect two-fold increase in number of copies per cycle). Relative telomere length (T/S ratio) was calculated using the mathematical model for relative quantification by Pfaffl [44]. In this model, the T/S ratio of the target amplicon (telomere repeats) is calculated based on E and the Cq difference (ΔCq) between the calibrator sample and the focal sample, and expressed in comparison to the reference amplicon (actin), following the formula:

$$ T/S\; ratio={\left({E}_{target}\right)}^{\Delta Cq\  target}/{\left({E}_{reference}\right)}^{\Delta Cq\  reference} $$

This model results in more reliable and exact estimates [44] of relative telomere length, since it does not presume optimal and identical PCR efficiencies E = 2 for the target and reference amplifications (as is the case for the commonly used “ΔΔCq” or Livak method; [45]).

The qPCR method, like several other techniques for estimating telomere length, detects telomeric repeats at both chromosomal ends and interstitial locations (reviewed in [46]). Interstitial telomeric repeats have been detected in many mammals and birds, but not in fishes of the order of Cypriniformes or Salmoniformes [47]. If present, the amount of interstitials varies between individuals [48], which may cloud relationships when comparing telomere length across individuals. The present study focused on telomere attrition rate instead of telomere length, thereby avoiding any confounding effects of interstitial sequences. The within-individual change of telomere length over time, as examined here, only measures the shortening of the terminally located telomeric repeats, because internally-located interstitials are unaffected by factors that shorten terminally located telomere repeats [48].

### Data processing and statistical analyses

We used a paired test design to investigate the within-individual change in relative telomere length (hereafter TL), separately for transgenic and wild-type half-sibs. When examining the effect of growth on telomere attrition, we selected for analysis the fin that had not been clipped initially to avoid any effects of regeneration on TL (comparison 1; Fig. 1). When analysing the effect of fin regeneration, however, we compared the TL of the two simultaneously sampled pelvic fins of each fish (during the second sampling event), one previously unclipped and one regenerated (comparison 2; Fig. 1).

For logistic reasons, we were not able to replicate the study beyond the level of the individual fish, or sample fish from specific families (as stated above). However, given the enormous difference in growth between the genotypes, it seems likely that any tank or family effects would be marginal in comparison. Consistently, a recent study of coho salmon showed that the variation in size between wild-type and GH-transgenic genotypes is many-fold larger than the variation between families within each genotype (Additional file 1: Table S1 in [49]).

For all analyses, we assumed that telomere data were approximately normally distributed, which was largely supported by Shapiro-Wilk tests. Thus, only one of six such tests resulted in a significant deviation from normality (pooling all data for transgenic fish; *W* = 0.96, *p* = 0.02). In contrast, examining TL separately for transgenic and wild-type fish during growth and regeneration, respectively, as well as pooling all data for wild-type fish, did not indicate a strong deviation from normality (*W* ≥ 0.93, *p* ≥ 0.06). Moreover, it should be noted that non-parametric tests yielded qualitatively similar results (not shown). We present means ± SE, varying sample sizes in the analyses are due to missing values.

## Results

**Fig. 2:**
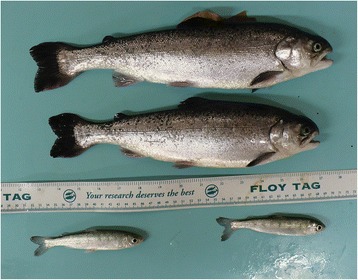
Morphological comparison between same age GH-transgenic and wild-type coho salmon. The picture was taken at second sampling, when the average transgenic fish (above ruler) had grown 7.1 times in length and 54.2 times in weight compared to the average wild-type coho salmon (below ruler)

After 10 months growth, GH transgenesis had resulted in on average 54-fold heavier and 7-fold longer fish compared to wild-type maternal half-sibs. Thus, mean length and weight gain for wild-type fish were 3.8 ± 0.7 cm (*n* = 14) and 13.5 ± 1.4 g (*n* = 15), while the corresponding values for transgenic fish were 25.5 ± 1.1 cm and 731.1 ± 58.6 g (*n* = 23; Fig. 2).

TL in wild-type fish ranged between 0.532 and 1.0564 (*n* = 15), and between 0.614–1.585 in transgenics (*n* = 23) [50]. On average, wild-type fish had shorter telomeres than transgenics on both sampling occasions (two-sample t-test, initial sampling: *p* < 0.001, final sampling: *p* = 0.005).

**Fig. 3:**
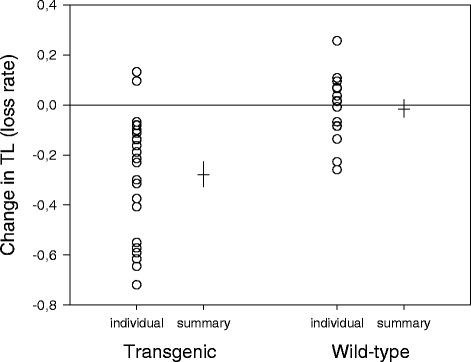
Telomere length (TL) change in pelvic fins of GH-transgenic and wild-type coho salmon. The graph shows individual data values as well as summary statistics (mean: horizontal line, SE: vertical line) of the change in telomere length between the sampling events. Each fish was sampled at the beginning and at the end of the 10 months’ period (see also Fig. 1, comparison 1). A negative value denotes telomere shortening during the growth period. Sample sizes were 23 (transgenic) and 15 fish (wild-type), respectively

**Fig. 4:**
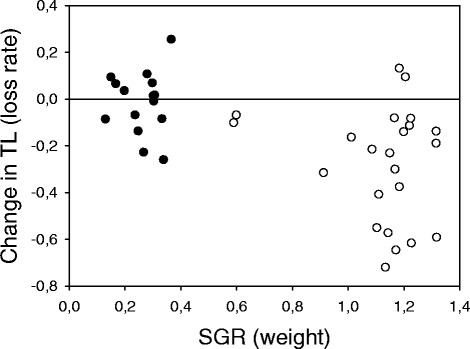
Specific growth rate (SGR) and change in telomere length (TL) in wild-type and GH-transgenic coho salmon. On average, the weight-based SGR per day of transgenics (open circles, *n* = 23) was 4.3 times higher than in wild-types (filled circles, *n* = 15). The reference line denotes no change in telomere length during the 10 months growth period of an individual. See Additional file 1 for calculations of SGR

In the fast-growing transgenic fish, telomeres shortened substantially, on average 24.1 %, while only a relatively small change was observed in the wild-type fish, 1.9 %. Repeated measures of fin TL thus revealed a substantial loss of telomeres in transgenic fish (paired t-test, *t* = –5.51, *p* < 0.0001), whereas no significant change was found in fish growing at a wild-type rate (*t* = –0.41, *p* = 0.69; Fig. 3). The effect of the genotype on telomere loss rate is further illustrated in Fig. 4.

**Fig. 5:**
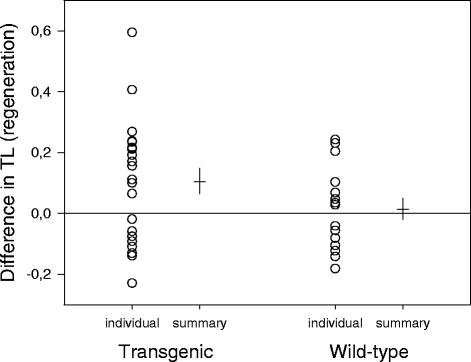
Effect of fin regeneration on telomere length (TL) in GH-transgenic and wild-type coho salmon. The graph shows individual data values as well as summary statistics (mean: horizontal line, SE: vertical line) of the difference in telomere length between the two simultaneously sampled pelvic fins of each fish, one previously unclipped and one regenerated (see also Fig. 1, comparison 2). A positive value indicates longer telomeres in the regenerated fin. Sample sizes were 22 (transgenic) and 15 fish (wild-type), respectively

Fin regeneration appeared to have a positive effect on TL in transgenic fish. Comparing TL in both pelvic fins clipped on the same sampling occasion, we found that telomeres in the regenerated fin were longer than in the equivalent fin that had not previously been clipped (paired t-test, *t* = 2.50, *p* = 0.021). In contrast, there was no significant difference in TL between regenerated and unscathed fins of wild-type coho (*t* = 0.43, *p* = 0.67; Fig. 5).

## Discussion

By analysing individuals repeatedly, we found a 12 times faster rate of telomere shortening in our sample of GH-transgenic coho salmons compared to their wild-type maternal half-sibs. Given the technical challenges and regulatory requirements when working with transgenic fish or other GMOs ([37] and references therein), we were not able to assess whether the difference in telomere loss rate between the two genotypes and their associated growth trajectories was influenced by other factors such as family or tank effects. As argued above, however, it seems likely that any such effects would be relatively small given the huge difference in growth between the genotypes (Fig. 2).

We did not detect any significant loss of telomeres in the wild-type fish (nor did we find a significant difference in TL between their regenerated and unscathed fins). Due to relatively small sample and effect sizes, however, we had low statistical power in these tests (<20 %) and can therefore not rule out that a larger sample might reveal a minor shortening of telomeres in these fish. Even so, our results demonstrate a striking difference in telomere erosion rate between the two genotypes (Figs. 3 and 4).

At both sampling occasions, transgenic fish had longer telomeres than wild-type fish. Our current data do not allow an investigation of possible reasons for this pattern. It might be explained by founder effects as TL is largely heritable across a taxonomically wide range of investigated species (e.g. [51–53]). Further work is required to determine the reasons for the difference in TL, as well as the generality of our results on telomere loss rate. Nevertheless, the striking difference in telomere attrition between transgenic and wild-type fish seems consistent with a trade-off between fast growth and somatic maintenance. Interestingly, Näslund et al. [41] recently presented results supporting that rapid growth induces costs in terms of reduced telomere maintenance in a related species, the brown trout. Below, we address the causes and consequences of telomere attrition in our model system.

### Mechanisms of telomere shortening during rapid growth

Rapid growth due to e.g. the overexpression of growth hormone requires high metabolic rates and thus increased aerobic respiration [54–56]. As by-products of oxidative metabolism, ROS are generated in mitochondria [8, 9]. In mammals, ROS production was found to be proportional to levels of growth hormone in GH-transgenic mice [7] and Ames dwarf mice, which naturally lack GH [57]. Taken together, these findings suggest that rapid growth can be associated with elevated levels of ROS (but see [58]).

If ROS production exceeds the cell’s antioxidant capacity, the resulting oxidative stress damages DNA and other macromolecules and has therefore been implicated in cellular senescence [9, 10]. Two recent studies of birds support a link between fast growth, oxidative damage, and telomere shortening [59, 60].

In our study, the rapid growth of transgenic fish was associated with substantial telomere loss per time unit. One possible explanation for this pattern is elevated oxidative damage to DNA mediated by ROS, as outlined above. Indeed, in two previous investigations, the exact same strain of GH-transgenic coho salmon (M77) upregulated their anti-oxidant defence system, showed higher levels of parameters related to oxidative stress, and more oxidative damage to proteins compared to wild-type fish [61, 62].

Telomeres are also somewhat shortened every time a cell replicates its DNA before division, i.e. during growth (see [19]). Thus, instead of ROS, a larger number of cell divisions may explain why more telomere repeats have been lost in transgenic fish compared to wild-type of the same age. It is, however, worth noting that two previous studies of the same strain of transgenic coho salmon found increased ROS damage independently of the achieved length of the fish (since also size-matched genotypes were compared; [61, 62]). In the present study, the change in TL per cm grown was on average 7 times higher in transgenic fish compared to wild-type fish (wild-type: -0.0016 cm^−1^ ± 0.011, *n* = 14; transgenic, -0.011 cm^−1^ ± 0.0018, *n* = 23). Moreover, despite their larger size, transgenic fish had longer telomeres than wild-type at both sampling occasions. It therefore seems unlikely that a larger number of cell divisions alone can explain the faster loss rate of telomeres in growth-enhanced transgenic fish.

### Possible implications of shortened telomeres

Studies on a broad range of species suggest that TL may serve as a fitness indicator, correlating with an individual’s longevity and/or reproductive success (e.g. [16, 20, 29, 63]). In GH-transgenic coho salmon, the accelerated loss of telomeres (this study) and increased levels of oxidative stress [61, 62] may be associated with their advancement in stage at age and markedly compressed lifespan [6]. Consistently, fast growth in GH-transgenic mice is accompanied by elevated stress hormone levels (plasma corticosterone), severely shortened lifespan (often more than 50 %), and reduced replicative potential of their cells when grown *in vitro* (reviewed in [64]), indicating telomere degradation. There is, however, an ongoing debate about the impact of oxidative stress on organismal ageing. For example, Perez et al. [65] found little effect of oxidative stress on the lifespan of mice, in which the expression of anti-oxidant enzymes was manipulated.

Transgenic fish show decreased survival for several reasons (e.g. [37, 66, 67]). One of them is reduced disease resistance, which may be linked to an inability to maintain telomeres (cf. [68]). While GH-transgenic coho salmon are not more susceptible to bacterial pathogens than wild-type fry, they suffer higher mortality compared to non-transgenics when infected at a later stage [69, 70]. Furthermore, fast-growing GH-transgenic mice show impaired T-cell responses [71]. An effective immune response relies on the ability of naïve lymphocytes to undergo massive cell divisions (reviewed in [72]), which requires sufficiently long telomeres. Consistently, naïve T-cells were found to have longer telomeres compared to differentiated memory cells [72]. Further support for an association between immune function and telomere attrition comes from studies of human genetic disorders. For example, patients with *dyskeratosis congenita* suffer from infections as well as dramatically shortened lymphocyte TL [72].

### Maintenance of telomere length under natural growth rates

Another interesting result of the present study was that wild-type coho salmons appeared better able to maintain TL than their transgenic half-sibs. Consistently, a recent experimental study on a natural population of brown trout found that juveniles were able to maintain TL during their second year of life [41]. Cells in highly proliferative tissues as well as “immortal” cells such as germ, stem, and tumour cells usually express telomerase to prevent the loss of telomeric DNA at chromosomal ends [25, 73]. Consistent with their ability for indeterminate growth, all investigated fish species to date were found to have high levels of telomerase activity even in somatic cells (reviewed in [31]). Given the striking difference in telomere maintenance ability between the two genotypes found here, future investigations of telomere dynamics in GH-transgenic fish should include a comparison of telomerase expression and activity levels between transgenic and wild-type half-sibs.

### Effects of fin regeneration on telomere length

Some species have retained the ability to regenerate injured tissues, as well as entire organs and limbs (reviewed in [34]). Studies of fish have documented an upregulation of telomerase expression in regenerating rayed fins, enabling TL in repeatedly clipped fins to be maintained [32, 33]. We compared TL in clipped and unclipped fins sampled at the same time and found, as expected, a significant difference only in the transgenic fish. Note that longer telomeres in regenerated fins do not necessarily imply an elongation of telomeres due to telomerase, but may indicate that the newly outgrown fin tissue was generated from stem cells [34], which have maintained the fish’s initial (longer) TL. Since wild-type fish seemed able to maintain their somatic telomeres while growing at natural rates, a re-setting of telomeres to their initial length after regeneration results in no detectable difference between naïve and re-grown fins. Although we presently cannot distinguish between the mechanisms that led to longer telomeres in regenerated transgenic fins, the result indirectly supports our hypothesis that fast growth is traded off against the ability to maintain TL in somatic cells.

## Conclusions

We show that enhanced growth is associated with accelerated telomere loss in a growth-manipulated fish model, while the wild-type maternal half-sibs, growing at natural rates, appeared able to maintain their telomeres. Our findings are consistent with the hypothesized evolutionary trade-off between rapid growth and somatic maintenance, and support long-standing theories of ageing.

### Availability of supporting data

The data set supporting the results of this article is available in the Dryad Digital Repository [50].

## Additional files

Additional file 1:
**Weight, length, and specific growth rate (SGR) of wild-type and GH-transgenic coho salmon.** Morphological characteristics of wild-type and GH-transgenic coho salmon at first (age ca. 7 months) and second sampling (age ca. 17 months). SGR is calculated for weight or length, respectively. (PDF 20 kb) Additional file 2:
**Summary of primer sequences used in telomere (Tel) and beta-actin (Actin) qPCR assays.** The primer name, its nucleotide sequence as well as the source that provided the primer sequence is presented. (PDF 42 kb) Additional file 3:
**Summary of standard curve characteristics for all telomere and beta-actin qPCR assays.** On each of five telomere and five beta-actin qPCR amplification plates, a standard curve was included in triplicate. (PDF 28 kb)
