# Supplementary material for: Rapid growth accelerates telomere attrition in a transgenic fish
Source: BMC Evol Biol. 2015 Aug 14;15:159. doi: 10.1186/s12862-015-0436-8 (PMC4535669; doi:10.1186/s12862-015-0436-8)
Supplement: Additional file 1: — Weight, length, and specific growth rate (SGR) of wild-type and GH-transgenic coho salmon. Morphological characteristics of wild-type and GH-transgenic coho salmon at first (age ca. 7 months) and second sampling (age ca. 17 months). SGR is calculated for weight or length, respectively. (PDF 20 kb) [file 12862_2015_436_MOESM1_ESM.pdf]

Additional file 1

Weight, length, and specific growth rate (SGR) of wild-type and GH-transgenic coho salmon. Morphological characteristics of wild-type (WT, n = 15) and GH-transgenic coho salmon (T, n = 23) at first (age ca. 7 months) and second sampling (age ca. 17 months). SGR is calculated for weight or length, respectively, as SGR = 100*[ln (second) – ln (first)] / 307 days.
Mean ± SE (first rows) and range (second rows) are presented.

	Weight (g)			Length (cm)		
	first	second	SGR	first	second	SGR
WT	12.0 ± 0.7	24.5 ± 1.7	0.26 ± 0.019	9.4 ± 0.2	13.2 ± 0.7	0.11 ± 0.015
	8.1 – 18.6	17.1 – 41.9	0.13 – 0.37	8.5 – 11.0	10.0 – 21.1	0.027 – 0.28
T	21.7 ± 0.5	752.8 ± 58.7	1.12 ± 0.039	12.1 ± 0.1	37.6 ± 1.1	0.37 ± 0.011
	17.2 – 26.9	136.0 – 1197.0	0.59 – 1.32	11.0 – 13.1	23.0 – 42.5	0.20 – 0.43
