# Supplementary material for: Rapid growth accelerates telomere attrition in a transgenic fish
Source: BMC Evol Biol. 2015 Aug 14;15:159. doi: 10.1186/s12862-015-0436-8 (PMC4535669; doi:10.1186/s12862-015-0436-8)
Supplement: Additional file 2: — Summary of primer sequences used in telomere (Tel) and beta-actin (Actin) qPCR assays. The primer name, its nucleotide sequence as well as the source that provided the primer sequence is presented. (PDF 42 kb) [file 12862_2015_436_MOESM2_ESM.pdf]

Additional file 2

Summary of primer sequences used in telomere (Tel) and beta-actin (Actin) qPCR assays**.** The primer name, its nucleotide sequence as well as the source that provided the primer sequence is presented.

Primer	Sequence (5′→ 3′)	Designed by
Tel 1b (forward)	CGG TTT GTT TGG GTT TGG GTT TGG GTT TGG GTT TGG GTT	R.M. Cawthon*
Tel 2b (reverse)	GGC TTG CCT TAC CCT TAC CCT TAC CCT TAC CCT TAC CCT	R. M. Cawthon*
Actin (forward)	TGG CAT CAC ACC TTC TAC	B. Wassmur†
Actin (reverse)	AAT CTG GGT CAT CTT CTC C	B. Wassmur†

*Eccles Institute of Human Genetics, Univ. Utah, USA; †Department of Biological and Environmental Sciences, Univ. Gothenburg, Sweden
