# Supplementary material for: Rapid growth accelerates telomere attrition in a transgenic fish
Source: BMC Evol Biol. 2015 Aug 14;15:159. doi: 10.1186/s12862-015-0436-8 (PMC4535669; doi:10.1186/s12862-015-0436-8)
Supplement: Additional file 3: — Summary of standard curve characteristics for all telomere and beta-actin qPCR assays. On each of five telomere and five beta-actin qPCR amplification plates, a standard curve was included in triplicate. (PDF 28 kb) [file 12862_2015_436_MOESM3_ESM.pdf]

Additional file 3

Summary of standard curve characteristics for all telomere and beta-actin qPCR assays**.** On each of five telomere and five beta-actin qPCR amplification plates, a standard curve was included in triplicate. For the telomere and beta-actin assays separately, the mean ± SE (first rows), as well as the range (second rows) are presented for the slope, y-intercept, coefficient of determination R^2, and PCR efficiency.

	Telomere	Beta-actin
Slope	-3.17 ± 0.024	-3.47 ± 0.085
	-3.24 – -3.098	-3.70 – -3.30
y-intercept	12.80 ± 0.019	24.16 ± 0.24
	12.73 – 12.84	23.68 – 24.93
R^2	0.998 ± 0.000316	0.992 ± 0.00261
	0.997 – 0.999	0.985 – 0.998
PCR efficiency	2.069 ± 0.012	1.95 ± 0.029
	2.034 – 2.10	1.87 – 2.010
